# Guillain-Barré Syndrome after Chikungunya Infection

**DOI:** 10.3201/eid1503.071482

**Published:** 2009-03

**Authors:** Gaëtan Lebrun, Karim Chadda, Anne-Hélène Reboux, Olivier Martinet, Bernard-Alex Gaüzère

**Affiliations:** Centre Hospitalier Felix Guyon, Saint-Denis, La Réunion, France

**Keywords:** Chikungunya infection, Guillain-Barré syndrome, Aedes albopictus, letter

**To the Editor:** *Chikungunya virus* is an RNA alphavirus (group A arbovirus) in the family *Togaviridae*. The known vectors are *Aedes aegypti* and *Ae. albopictus* mosqitoes. Chikungunya infection, after an incubation period of 2–10 days, has the main clinical manifestations of fever, polyarthralgia, and rash. Treatment consists of rest and medication for pain. Outcome is marked by incapacitating arthralgia, which can persist for several weeks or months (*1*). Complications are rare and consist of mild hemorrhage, myocarditis, and hepatitis (*2*). Neurologic manifestations are less well known (*3*). Infection is confirmed by the identification of genomic products in acute-phase blood specimens, (reverse transcription–PCR [RT-PCR]) or, more recently, by serum immunoglobulin (Ig) M or a 4-fold increase in other antibodies. In 2006, chikungunya virus was found on Réunion Island; seroprevalence on the island was estimated to be 38.2% among 785,000 inhabitants (95% confidence interval 35.9%–40.6%) (*4*).

Guillain-Barré syndrome (GBS) is an acute inflammatory demyelinating polyneuropathy; incidence worldwide is 0.6–4/100,000 persons/year. In two thirds of patients, neuropathic GBS occurs after an infection (*5*,*6*).

Cases of GBS have been described in association with the arboviruses dengue and West Nile but not with chikungunya virus. We report 2 cases of acute and severe GBS related to infection with chikungunya virus.

The first patient was a 51-year-old woman who in 2006 was admitted to an intensive care unit in Réunion Island’s Centre Hospitalier Departemental for treatment of polyradiculoneuropathy. Her medical history consisted of poorly treated type 2 diabetes and hypertension. Three weeks before hospital admission, she had had fever, arthralgia, rash, and diarrhea. One week later, rapidly progressing motor weakness and sensory disturbances developed, e.g., tingling in all limbs. She had facial diplegia, and her tendon reflexes were absent. Cerebrospinal fluid (CSF) contained increased protein (1.44 g/L) but not increased leukocytes (1/mm^3^). Electromyography displayed typical signs of demyelinating sensorimotor neuropathy with increased distal motor latency and reduced motor conduction velocity. Sensory nerve action potential was absent. Antichikungunya IgM was found in serum at 15 days after onset of signs and symptoms. This seroconversion confirms an acute infection by an alphavirus. Serum genomic product (RT-PCR, TaqMan method) (*7*) was negative for chikungunya virus. Antichikungunya IgM and IgG were also found in CSF.

The patient’s respiration rapidly deteriorated, and she required tracheal intubation and mechanical ventilation for 12 days. She was given intravenous immunoglobulin for 5 days (TEGELINE; LBF Biomedicaments, Courtaboeuf, France). She recovered and was extubated on day 12. Two months after onset of symptoms, the patient reported a satisfactory recovery; she was able to walk, and her sensory disturbances had rapidly disappeared.

The second patient was a 48-year-old woman who in 2006 was admitted to the intensive care unit in Réunion Island’s Centre Hospitalier Departemental unit for a rapidly developing polyradiculoneuropathy. She had no relevant past medical history. Two weeks before her admission, she had been febrile and had had arthralgia and a rash. Later, weakness with facial diplegia and sensory disturbances developed, e.g., tingling in all limbs. Tendon reflexes were absent. CSF contained increased protein but not increased leukocytes. Electromyography displayed signs of a peripheral neuropathy and evidence of a conduction block. At the time of hospital admission, antichikungunya IgM and IgG were detected in 2 serum samples. RT-PCR for chikungunya virus in serum and CSF was negative.

The patient’s respiration rapidly deteriorated, and she required tracheal intubation and mechanical ventilation for 9 days. After receiving intravenous immunoglobulin for 5 days, she recovered quickly. Return of a productive cough and satisfactory muscle tone enabled her to be removed from mechanical ventilation on day 9.

For the 2 patients reported here, GBS diagnosis was based on a typical clinical acute motor and sensory polyradiculoneuropathy, which evolved in 3 characteristic stages: rapid deterioration, plateau, and slow recovery (*6*). Also typical of GBS are normal CSF counts, increased CSF proteins, and electromyography data (peripheral neuropathy, conduction block). The widespread screening for organisms known to be associated with GBS produced negative results. However, antichikungunya IgM was found in serum and CSF, although genomic products in serum and CSF were negative, which was not surprising, given the brief period (4–5 days) of viremia (*8*). These findings strongly supported a disseminated acute chikungunya infection and enabled us to conclude that chikungunya virus was probably responsible for the GBS.

Epidemiologic data also support a causal relationship between chikungunya infection and GBS. The incidence rate of GBS increased ≈22% in 2006 (26/787,000 [3.3/100,000] persons) over the rate in 2005 (21/775,000 [2.7/10,000] persons) and then declined to a rate closer to baseline in 2007 (23/800,000 [2.87/100,000] persons).

These 2 cases of GBS on Réunion Island were related to an acute and documented chikungunya infection. In the absence of an effective treatment, patients with these suspected infections should receive supportive care for classic GBS.
